# Exercise as an intervention for first‐episode psychosis: a feasibility study

**DOI:** 10.1111/eip.12329

**Published:** 2016-03-14

**Authors:** Joseph Firth, Rebekah Carney, Rebecca Elliott, Paul French, Sophie Parker, Rebecca McIntyre, Jamie S. McPhee, Alison R. Yung

**Affiliations:** ^1^ Institute of Brain, Behaviour and Mental Health University of Manchester Manchester UK; ^2^ Manchester Academic Health Sciences Centre University of Manchester Manchester UK; ^3^ Greater Manchester West NHS Mental Health Foundation Trust Manchester UK; ^4^ Department of Psychological Sciences The University of Liverpool Liverpool UK; ^5^ School of Psychological Sciences University of Manchester Manchester UK; ^6^ Lincolnshire Partnership NHS Foundation Trust Lincolnshire UK; ^7^ School of Healthcare Science Manchester Metropolitan University Manchester UK

**Keywords:** early intervention, exercise, physical activity, physical health, schizophrenia

## Abstract

**Aim:**

Exercise can improve psychiatric symptoms, neurocognitive functioning and physical health in schizophrenia. However, the effects in early psychosis have not been explored. This study aimed to assess the feasibility of an exercise intervention for early psychosis and to determine if it was associated with changes in physical and mental health.

**Methods:**

Thirty‐one patients with first‐episode psychosis (FEP) were recruited from early intervention services to a 10‐week exercise intervention. The intervention group received individualized training programmes, aiming to achieve ≥90 min of moderate‐to‐vigorous activity each week, using exercise programmes tailored to individual preferences and needs. A comparison FEP sample from the same services (*n* = 7) received treatment as usual.

**Results:**

Rates of consent and retention in the exercise group were 94% and 81%, respectively. Participants achieved an average of 107 min of moderate‐to‐vigorous exercise per week. Positive and Negative Syndrome Scale total scores reduced by 13.3 points after 10 weeks of exercise, which was significantly greater than the treatment as usual comparison group (*P* = 0.010). The greatest differences were observed in negative symptoms, which reduced by 33% in the intervention group (*P* = 0.013). Significant improvements were also observed in psychosocial functioning and verbal short‐term memory. Increases in cardiovascular fitness and processing speed were positively associated with the amounts of exercise achieved by participants.

**Conclusion:**

Individualized exercise training could provide a feasible treatment option for improving symptomatic, neurocognitive and metabolic outcomes in FEP.

## Introduction

Exercise has been found to reduce positive and negative symptoms and improve cognitive functioning in people with long‐term schizophrenia, provided that sufficient amounts of moderate‐to‐vigorous activity are achieved.^1^ However, this has yet to be explored in first‐episode psychosis (FEP), despite evidence that interventions to improve negative and cognitive symptoms may be more effective in the early phases of illness.^2^, ^3^ Furthermore, healthy lifestyle interventions, which incorporate both exercise and dietary components, can significantly improve psychosocial functioning in FEP.^4^ Cross‐sectional studies have also indicated that physical activity and fitness are associated with improved functional outcomes.^5^, ^6^

Additionally, FEP is a critical period for attenuating weight gain and metabolic dysfunction, which begins within weeks of starting antipsychotic medication.^7^, ^8^ There is a fivefold increase in metabolic syndrome within the first few years of treatment,^9^ which heightens the risk of cardiovascular diseases and contributes towards the premature mortality of 15–20 years for people with schizophrenia.^10^ Physical activity interventions may counteract this, because cardiorespiratory fitness is one of the strongest predictors of metabolic health following anti‐psychotic treatment,^11^ and is improved by exercise during the early stages of illness.^12^, ^13^ FEP may also be the optimal timeframe to establish lasting habits of regular exercise when patients are younger, more active^14^ and relatively free of obesity‐related disorders, which may act as a barrier towards exercise.^15^

We conducted an exploratory study, ‘Investigating the Benefits of Exercise in Early Psychosis’ (‘IBEEP’) to examine the extent to which young adults with FEP will participate in exercise and to assess any associated change in symptoms, cognitive functioning and physical health. Previous exercise interventions in schizophrenia have had poor adherence and high attrition,^16^ possibly due to low motivation among patients.^12^, ^17^ Thus, we used individualized exercise programmes, tailored to each participant's preferences and needs, to make exercise accessible and intrinsically motivating.

## Method

The IBEEP study was approved by the North West Research Ethics Committee on 18/12/2013 (REC# 13/NW/0784) and registered with the current clinical trials database (ISRCTN09150095). Participants were recruited from community‐based early intervention (EI) services in Greater Manchester West NHS Trust. In the United Kingdom, EI services are offered to any individuals aged 14–35 who are experiencing FEP (defined as full‐threshold psychotic symptoms for a period of greater than 7 consecutive days), regardless of formal diagnostic status. Thus, in order to assess exercise as an intervention for FEP more broadly and inform its implementation within EI services, no restrictions were placed on patients’ diagnosis in this study. Inclusion criteria for the intervention group were the following: (i) being a current service user of EI services within the first 5 years of a psychotic disorder; (ii) aged 18–35; (iii) experiencing current psychological difficulties, having either a score of ≥2 on the World Health Organization (WHO) Disability Assessment Schedule 2.0 (WHODAS 2.0)^18^ or ≥21 on the Beck Depression Inventory 2.0 (BDI‐II).^19^ Exclusion criteria were the inability to provide informed consent, pregnancy, insufficient English language to complete assessments, and/or physical health issues that contraindicated exercise. These included poorly controlled asthma, diagnosed heart conditions, untreated hypertension and other medical conditions, and were assessed by the referring clinician.

Recruitment was conducted by meeting with EI teams and asking them to refer any of their service users who seemed suitable to participate in the study (along with leaving leaflets in EI waiting rooms). Those referred were contacted by telephone to assess their interest in the study and then screened against the inclusion criteria before being met in person to provide written informed consent. The study was conducted as a non‐randomized feasibility trial, with all participants being allocated to the intervention group. Comparison data were obtained from the treatment‐as‐usual (TAU) control group of a separate trial, which was taking place within the same clinical services over the same timeframe (ISRCTN06815355). These participants were also in the first 5 years of onset of a psychotic disorder and aged 18–35 but received no exercise.

### Intervention

Exercise was delivered through community leisure services. These services are widespread in UK and are funded by local government to offer subsidized gym memberships and sporting activities for people referred by the National Health Service. The intervention was 10 weeks long and designed to enable each participant to achieve 90 min of moderate‐to‐vigorous exercise per week, as this amount of exercise can improve physical and mental health in schizophrenia.^1^

Participants were offered gym training sessions twice per week at their local leisure centres, supervised by a research assistant who had several years of exercise experience. A standardized training guide (Supporting Information) was developed through consultation with the exercise physiologist (JM). Sessions aimed to achieve 45–60 min of moderate‐to‐vigorous activity using aerobic and resistance exercises. The specific activities for any given session were selected on the basis of participant preference, in order to maximize adherence and engagement with exercise.^20^ Options for aerobic exercise to improve cardiovascular health included treadmills, cycle ergometers and rowing machines. Resistance training included exercises for all the major muscle groups of the arms, legs and torso to increase muscle tone and strength. For resistance exercises, participants completed three sets of 8–12 repetitions per exercise. Mandatory gym inductions were provided by personal trainers at community leisure centres (who were also available for further support throughout).

Additionally or alternatively to the gym training sessions, participants were able to undertake other sporting activities to meet their weekly exercise targets (Table 1). Research assistants took responsibility for arranging participants’ access and transport to gyms/leisure services. An exercise logbook was used to set initial goals and monitor participants’ improvement during the intervention, both of which can increase motivation and adherence.^21^ The logbook was also completed by research assistants after each session to record attendance, duration, modality and intensity of exercise. This standardized process was replicated across all three EI sites.

**Table 1:** Popularity of different exercise options

Exercise type	Service users taking part, *n* (%)	% of all exercise sessions recorded
Gym sessions: supervised	27 (96)	65.5
Gym sessions: alone	14 (50)	13.3
Football	2 (11)	9
Boxing/martial arts	3 (11)	5
Cycling	2 (7)	4
Badminton	3 (7)	3
Swimming	3 (11)	2
Gym: fitness classes	3 (11)	>1

### Primary outcomes

As a feasibility trial, the primary outcomes were recruitment, retention and amount of exercise achieved. Recruitment rate was defined as ‘number of patients who consented to take part’ divided by ‘number of patients approached’. Retention was defined as completing both the 10‐week intervention and the psychiatric assessments at follow‐up. The amount of exercise achieved was determined from the logbooks, calculated as average minutes per week (mean) over 10 weeks. This was analysed across all participants who were offered the intervention to provide a realistic evaluation of how the intervention would transpire in practice. The target amount of exercise was set at 90 min of moderate‐to‐vigorous activity per week.^1^, ^22^ Any reported increases in low‐intensity exercise (i.e. walking) were also recorded, but analysed separately.

### Between‐subjects change outcomes

The principle change outcome was psychiatric symptoms. This was measured using the Positive and Negative Syndrome Scale (PANSS).^23^ This structured clinical interview was administered by research assistants to each participant during the week prior to commencing their intervention and then repeated within 2 weeks of completion (or within 8–12 weeks of the baseline assessments for the TAU group).

### Within‐subjects outcomes

A range of additional measures were administered to the intervention group to investigate which other outcomes could feasibly show significant change from 10 weeks of exercise, thus informing target outcomes for future RCTs. To reduce participant burden (and assess the acceptability of these assessments), subjects were able to opt in or out of secondary measures without affecting their status in the trial. Secondary measures of mental health included the BDI‐II^19^ for depression and the ‘Social Interaction Anxiety Scale’ 24 for anxiety. Functional disability was evaluated using the ‘Socio‐Occupational Functioning Assessment Scale’,^25^ the WHODAS 2.0^18^ and the WHO Quality of Life Brief Assessment (WHOQOL‐BREF).^26^

Physical health was assessed using standard procedures for body mass index, waist circumference and systolic/diastolic blood pressure. Aerobic fitness was estimated using the 6‐Minute Walk (6MW) test,^27^ conducted on a 20‐m track in a sports hall. Muscular power was assessed as ‘vertical jump height’ using the Sargent Jump protocol.^28^ Changes in self‐reported physical activity were measured using the International Physical Activity Questionnaire.^29^

Computerized cognitive testing was administered using a touch‐screen interface. Our battery consisted of four ‘Cambridge Neuropsychological Test Automated Battery’^30^ tasks, four traditional neuropsychological tasks (administered using ‘Psychology Experiment Building Language’^31^) and a test of social cognition. The full task battery is detailed in Table 2.

**Table 2:** Neurocognitive test battery

Cognitive domain	Task(s)	Software
Verbal short‐term memory	12‐word verbal recall	cantab
Processing speed	Trail Making Task A	pebl
	Trail Making Task B	pebl
	Digit‐Symbol Coding	pebl
Executive functioning	Stockings of Cambridge	cantab
	Spatial span	cantab
Inhibitory control	Erikson Flanker Task	pebl
Motor function	Finger tapping	pebl
	Motor screening	cantab
Social cognition	Mind in the eyes	flash

CANTAB, Cambridge Neuropsychological Test Automated Battery; PEBL, Psychology Experiment Building Language.

### Statistical analysis

Statistical tests were conducted in spss 20.^32^ Values were checked for normality using Shapiro–Wilk tests and normal probability plots. A ‘full analysis set’ approach was used throughout to include all available data for every outcome measure.^33^ Primary outcomes of feasibility were summarized using sample means and percentages. Changes in psychiatric symptoms were compared between exercise and TAU groups using between‐subjects *t*‐tests (Welch's *t*). Additional outcomes measures for the intervention group were assessed using within‐subjects *t*‐tests after applying a Bonferroni correction to account for the number of measures used.

## Results

### Feasibility measures

Within 5 months of recruitment (05/01/2014–06/06/2014), 51 service users had been referred by EI services. These were contacted in date order until the target sample of 30 was surpassed. The recruitment rate was 94%; 31/33 of eligible referrals who were contacted were willing to participate in the IBEEP study and subsequently consented to take part. . All were EI service users aged 18–35 (average length of service use = 1.9 years), currently receiving antipsychotic medications. In addition, comparison data were available from the seven EI service users who had been allocated to the TAU control group of a separate trial. Baseline characteristics are compared in Table 3.

**Table 3:** Baseline characteristics of participants

	Exercise group (*n* = 31)	Control group (*n* = 7)	Between‐groups *P* value
Gender			
Male; *n* (%)	25 (81)	5 (82)	
Female; *n* (%)	6 (19)	2 (18)	0.624
Age, years; mean (s.d.)	25.8 (4.6)	25.9 (5.9)	0.984
Time in EIS, years; mean (s.d.)	1.9 (1.4)	2.02 (1.5)	0.869
Diagnosis; *n* (%)			
Non‐organic psychosis	15 (48)	5 (71)	
Schizophrenia	9 (29)	1 (14)	
Schizoaffective disorder	3 (10)	0	
Bipolar disorder w/ psychotic features	1 (3)	1 (14)	
Other psychotic disorder	3 (10)	0	
Psychiatric Symptoms			
PANSS total; mean (s.d.)	79.0 (18.0)	71.9 (12.8)	0.326
PANSS positive; mean (s.d.)	18.9 (6.2)	18.4 (4.0)	0.858
PANSS negative; mean (s.d.)	19.0 (6.1)	14.7 (4.5)	0.185
PANSS general; mean (s.d.)	21.7 (10.9)	36.3 (7.3)	0.335

EIS, early intervention services; PANSS, Positive and Negative Syndrome Scale.

**Figure 1:**
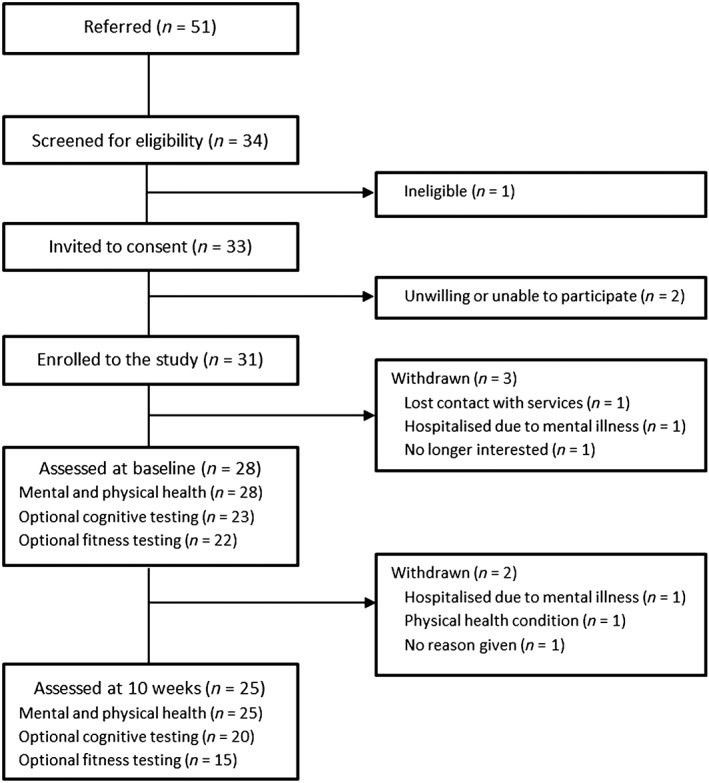
Flow chart of participants’ progress.

The retention rate was 81%, with 25/31 of the intervention group completing the 10‐week exercise programme and follow‐up assessments (Fig. 1). Three of the 6 dropouts were related to ‘serious adverse events’: one for chronic physical illness and two due to mental illness leading to hospitalization. These serious adverse events were reviewed by the NHS Research Office and deemed unrelated to study participation.

The average amount of moderate‐to‐vigorous exercise (as recorded in the logbooks) for the 28 participants who were offered the intervention was 107 min per week. Half of the participants exceeded the exercise target, averaging ≥90 min per week for 10 weeks, and 79% achieved ≥60 min per week. Sixteen participants also reported increasing their low‐intensity exercise during their exercise programme, achieving a mean average of 41.4 min of additional walking per week.

Supervised gym sessions accounted for the vast majority of exercise achieved by participants (Table 1). In line with participant preference, sessions mostly comprised of a mixture of aerobic and resistance exercises (Supporting Information) and were conducted either in a one‐to‐one basis or in small groups of two to three participants. Reasons given for missed sessions were physical ill health (27%), conflicting social/occupational commitments (26%), conflicting healthcare appointments (14%), feeling tired/aching (11%), medication side effects (8%) and mental ill health (6%).

### Between‐group comparisons

Total PANSS scores in the intervention group reduced by 13 points (27%) following 10 weeks of exercise. This was significantly greater than the 3.3 point reduction observed from TAU (*t* = 9.80, *P* = 0.010) (Table 4). PANSS subscales scores showed statistically significant benefits of exercise for negative symptoms and general symptoms, which reduced by 33% (*t* = 3.04, *P* = 0.013) and 25% (*t* = 4.73, *P* = 0.03), respectively. However, the 25% decrease in positive symptoms did not significantly differ from TAU (*t* = 2.06, *P* = 0.168).

**Table 4:** Symptomatic outcomes of exercise versus usual care

Exercise group (*n* = 25)				Control group (*n* = 7)			Between‐groups tests		
	Baseline (s.d.)	Follow‐up (s.d)	Change (s.d.)	Baseline (s.d.)	Follow‐up (s.d)	Change (s.d.)	Effect size (*g*)	*t*	*P* value
PANSS total	78.6 (17)	65.5 (12.2)	‐13.1 (14.9)	71.9 (12.8)	68.6 (13.6)	‐3.3 (5.2)	0.71	9.80	0.010*
PANSS subscales									
Positive symptoms	18.8 (5.9)	15.8 (5.7)	‐2.9 (3.5)	18.4 (4.0)	17.6 (5.8)	‐0.9 (3.2)	0.58	2.06	0.1680
Negative symptoms	19.2 (5.4)	15.1 (4.4)	‐4.0 (5.1)	15.7 (4.7)	14.7 (4.5)	‐1 (1.4)	0.64	3.04	0.013*
General symptoms	40.7 (8.4)	34.5 (5.1)	‐6.2 (8.8)	37.7 (7.1)	36.3 (7.3)	‐1.4 (2.9)	0.58	4.73	0.030*

PANSS, Positive and Negative Syndrome Scale.

* Statistically significant difference.

### Within‐group comparisons

Changes in additional outcome measures are displayed in Table 5. There was no significant improvement in depression (BDI‐II), anxiety (Social Interaction Anxiety Scale) or functional disability (WHODAS/WHOQOL). However, socio‐occupational functioning increased by 5.8 points on the Socio‐Occupational Functioning Assessment Scale’ scale (*P* < 0.001).

**Table 5:** Pre‐intervention and post‐intervention change in additional outcomes

Assessment	Baseline score (s.d.)	Follow‐up score (s.d.)	Effect size (*d*)	*t*‐value	*P*‐value
*Mental Health* (*n* = *25*)					
BDI‐II	22.3 (11.4)	18 (10.9)	0.38	1.92	0.067
SIAS	33.6 (17.8)	28.1 (14.7)	0.36	1.78	0.088
WHODAS	13.5 (8.0)	10.7 (7.1)	0.38	1.90	0.069
WHOQOL‐BREF^a^	81.6 (15.8)	85.6 (13.9)	‐0.26	‐1.28	0.213
SOFAS^a^	46.8 (8.1)	52.6 (10.1)	‐0.74	‐3.60	0.001*
*Physical Health* (*n* = *25*)					
BMI	30.2 (6.9)	29.8 (6.7)	0.23	1.14	0.265
Waist circumference (cm)	102.2 (16.1)	100.3 (16.6)	0.58	2.91	0.008
Systolic BP	128 (13.1)	123.2 (8.9)	0.47	2.04	0.057
Diastolic BP	80.3 (13.2)	77 (14.1)	0.22	0.97	0.343
IPAQ Mod/Vig METS^a^	247 (459)	942 (894)	0.95	4.55	<0.001*
*Cognitive Battery* (*n* = *20*)					
Verbal STM^a^	6.95 (1.3)	8.1 (1.6)	‐0.88	‐3.93	0.001*
Trail Making – A (seconds)	23.2 (7.0)	20.7 (4.2)	0.4	1.81	0.086
Trail Making – B (seconds)	33.4 (9.5)	29.5 (8.5)	0.65	2.9	0.009
Digit Coding^a^	26.1 (5.2)	26.8 (4.8)	‐0.17	‐0.78	0.444
Stocking of Cambridge^a^	7.3 (2.1)	8.1 (2.1)	‐0.48	‐2.17	0.043
Spatial Span^a^	5.6 (1.5)	5.65 (1.0)	‐0.07	‐0.34	0.741
Flanker conflict cost – accuracy	0.19 (0.2)	0.08 (1.0)	0.53	2.19	0.044
Flanker conflict cost – time	32.7 (39.9)	35.0 (27.7)	‐0.05	‐0.2	0.848
Finger tapping^a^	56.9 (9.9)	57.8 (10.5)	‐0.12	‐0.53	0.605
Motor Screening (secs)	0.85 (0.2)	0.86 (0.2)	‐0.06	‐0.26	0.799
Eyes task^a^	18.6 (4.7)	20.2 (5.9)	‐0.67	‐2.93	0.009
*Fitness testing* (*n* = *15*)					
6‐min walk distance (m)^a^	469.1 (73.8)	502.8 (80.8)	‐0.56	‐2.1	0.057
Vertical jump (cm)^a^	27.0 (10.1)	28.0 (8.9)	‐0.27	‐0.85	0.415

BDI‐II, Beck Depression Inventory 2.0; BMI, body mass index; BP, blood pressure; IPAQ Mod/Vig METS; International Physical Activity Questionnaire moderate/vigorous activity metabolic equivalents per‐week; SIAS, Social Interaction Anxiety Scale; SOFAS, Social and Occupational Functioning Assessment Scale; STM, short‐term memory; WHODAS, WHO Disability Assessment Scale 2.0; WHOQOL‐BREF, WHO Quality of Life Brief Assessment.

a Higher scores indicate improvement.

* Statistically significant at Bonferroni‐corrected threshold (*p* = 0.002)

Among the cognitive domains, verbal short‐term memory showed the greatest change, increasing from 6.2 to 8.1 words (*P* < 0.001). Moderate improvements in social cognition (‘Mind in the Eyes’, *P* = 0.009), processing speed (‘Trail Making‐A’, *P* = 0.086 and ‘Trail Making‐B’, *P* = 0.009), executive functioning (‘Stockings of Cambridge’, *P* = 0.043) and inhibitory control (‘Flanker task’, *P* = 0.044) fell short of the Bonferroni‐adjusted significance level. No changes occurred in motor function or spatial span.

In physical health, body mass index remained unchanged (−0.34, *P* = 0.265). Waist circumference decreased by 2 cm (*P* = 0.008), although this improvement also fell short of the adjusted significance threshold. Blood pressure showed no change in either systolic (−4.7 mmHg, *P* = 0.057) or diastolic (−3.37 mmHg, *P* = 0.343) readings. Fitness tests were completed by only 15 participants; the 34‐m increase in 6‐min walk distance was not statistically significant (*P* = 0.057), and there was no change in standing jump. Self‐reported activity levels (calculated from the International Physical Activity Questionnaire guidelines as moderate‐to‐vigorous MET‐minutes per week) had more than trebled by the end of the intervention (*P* = 0.001), even after excluding the one high‐end outlier. Additionally, this self‐reported activity correlated strongly with amounts of exercise recorded in logbooks (*rs* ^25^ = 0.717, *P* < 0.001).

### *Post hoc* testing

On the basis of previous literature, we had expected 6MW distance (fitness) and the Trail Making Task (processing speed) would be particularly sensitive to exercise. However, improvements in these areas did not reach significance, perhaps due to smaller numbers of participants completing these measures. Thus, a *post hoc* analysis was conducted to examine the relationship between these outcomes and engagement in exercise. The amount of exercise achieved was significantly correlated with improvements in fitness (*rs*(14) = 0.55, *P* = 0.042) and processing speed (Trail Making‐A (*rs*(20) = 0.48, *P* = 0.032), Trail Making‐B (*rs*(20) = 0.47, *P* = 0.037)).

## Discussion

### Feasibility of the intervention

Individualized exercise has previously been indicated as the optimal method for increasing physical activity among people with serious mental illness, as allowing participants to choose preferred activities can their boost intrinsic motivation towards exercise.^20^ Although previous exercise studies in schizophrenia have experienced poor adherence,^16^ our study found that young people, in the early stages of illness, engaged well with individualized exercise. Although participants were largely inactive upon entering the study (as indicated by baseline International Physical Activity Questionnaire scores^29^), participants changed their sedentary lifestyles to achieve 107 min of moderate‐to‐vigorous activity per week for 10 weeks, thus surpassing the 90‐min target.

Of 28 participants who received the intervention, 25 were retained over the 10‐week intervention and pre‐post assessments. This compares favourably with attrition observed in long‐term schizophrenia and even healthy populations.^16^, ^34^ The acceptability of exercise in EI services was also indicated by the ample number of referrals received from care coordinators, who were asked to refer any service users who might be suitable for the trial. Furthermore, a high proportion of those invited to who were invited to consent did participate in the study (94%). These findings suggest that individualized gym training is a feasible and engaging intervention in FEP.

### Exercise and mental health

This was the first study to examine the effects of exercise on psychiatric symptoms in early psychosis. Total PANSS scores decreased by 27% after 10 weeks of exercise; a significantly greater improvement than in the TAU group. The greatest improvements were observed in negative symptoms, which are often left untreated in FEP, but have a particularly strong impact on long‐term recovery.^35^, ^36^ The magnitude of improvement observed in both total symptoms and negative symptoms was clinically significant.^37^ We also found a significant improvement in social functioning in the intervention group. A blinded RCT is now required to definitively establish the efficacy of exercise for FEP.

Our findings are consistent with exercise studies in long‐term schizophrenia, showing that ≥90 min of moderate‐to‐vigorous exercise per week can significantly reduce psychiatric symptoms.^1^ Through providing effective treatment for negative symptoms early on, exercise could be used to facilitate full recovery and reduce the likelihood of enduring disability. Recent observational studies have similarly reported a positive association between exercise and better functional outcomes in FEP.^4^, ^5^, ^6^

Previous research in long‐term schizophrenia has shown that improving physical fitness may also reduce cognitive deficits, possibly through increasing brain‐derived neurotrophic factor.^38^, ^39^, ^40^ Deficits in verbal STM and processing speed are common in FEP and predictive of overall functioning.^41^, ^42^ Furthermore, these domains are the most sensitive to exercise in long‐term patients.^43^, ^44^, ^45^ In this study, verbal STM showed the largest pre‐post changes, exceeding the degree of improvement that can be accounted for by practice effects.^46^ Furthermore, increase in processing speed was positively associated with the amounts of exercise achieved, indicating a dose–response relationship in FEP.

### Exercise and physical health

People with schizophrenia are substantially more inactive than the general population,^47^ and this is associated with the obesity and cardiometabolic diseases observed in this population.^48^ The early stages of psychosis may be the optimal period for implementing exercise to improve physical health, because preventing cardiometabolic disorders from arising is more feasible than reversing the long‐term consequences, and patients are more likely to engage with physical activity at this time.^14^, ^49^

The 0.9 kg decrease in body weight and 2 cm reduction in waist circumference both fell short of significance. Nonetheless, these marginal improvements after just 10 weeks are encouraging findings, because changes in body composition generally require longer‐term exercise programmes.^50^ Furthermore, a more realistic and immediate goal of exercise programmes in FEP may be attenuating the usual trajectory of weight gain.^51^

Previous studies have also observed successful attention of anti‐psychotic induced weight gain from exercise in FEP, along with significant improvements in aerobic fitness, as measured by V02 Max (which is the gold standard in fitness testing).^4^, ^12^, ^13^ In this study, improvements in 6‐min walk distance (+34 m) fell short of statistical significance (*P* = 0.057). This may be due the relatively short training period or the assessment method used, because the 6MW test was originally developed for older adults.^27^ It therefore may not be sensitive to subtle changes in aerobic fitness in young people with psychosis.^52^ Nonetheless, a *post hoc* analysis revealed a correlation between increases in fitness and amounts of exercise achieved by participants, indicating a dose–response relationship. There was also a large increase in self‐reported physical activity, which had trebled after 10 weeks. If sustained, this would confer substantial health benefits for people with FEP, because fitness and physical activity are more predictive of all‐cause mortality than any other metabolic risk indicators.^53^, ^54^

### Limitations and conclusions

The findings of this exploratory study are limited by the non‐randomized design and small sample size, particularly in the control condition. A further limitation is the multifaceted nature of the intervention, which makes it difficult to separate the physiological effects of exercise from the positive social context within which it was administered. Future studies could overcome these limitations using randomized, time‐and‐attention control conditions.

It should also be considered that the research assistants who facilitated exercise had no formal exercise qualifications. Instead, the intervention was developed on the basis of recommendations from an exercise physiologist (JM), and on‐site exercise instruction was provided by qualified staff at community leisure centres. Nonetheless, the high levels of acceptability and engagement suggests that future trials could also take advantage of the expertise and facilities available through community leisure services to provide low cost and destigmatising interventions. Our findings indicate this could improve physical, psychological and social outcomes for young adults with FEP.^55^ Future work should therefore investigate novel methods for connecting EI services with exercise resources, and for embedding exercise specialists within EI services, in order to maximize accessibility and broaden possibilities for delivering such interventions in clinical practice.

## Supporting information


**Text S1** IBEEP Gym‐training guide.

Supporting info item
